# Associations Between Heart Rate Variability and Inflammation From Two Aging Studies: Racial and Age Differences

**DOI:** 10.1093/geroni/igaf122.3713

**Published:** 2025-12-31

**Authors:** Molly Wright, Richard Sloan, Carol Derby, David Almeida, Christopher Engeland

**Affiliations:** The Pennsylvania State University, University Park, Pennsylvania, United States; Columbia University Irving Medical Center, New York, New York, United States; Albert Einstein College of Medicine, Bronx, New York, United States; The Pennsylvania State University, University Park, Pennsylvania, United States; The Pennsylvania State University, University Park, Pennsylvania, United States

## Abstract

The vagus nerve regulates inflammation via a cholinergic anti-inflammatory pathway, supported by findings of a negative association between high-frequency heart rate variability (HF-HRV)—a marker of cardiac vagal activity—and inflammation. This study sought to examine whether there are racial and age differences in this association among mid-life and older adults. In the Einstein Aging Study, 74 adults (Mage=78.3; 45.9% Black) wore ambulatory electrocardiogram (ECG) devices for 7 days to measure HF-HRV and provided blood samples for inflammatory markers, including tumor necrosis factor (TNF)-α. Covariate-adjusted regression models showed HF-HRV was negatively associated with TNF-α (β=–0.323, p = 0.005); this was apparent in Black participants (β=–0.469, p = 0.008) but not White participants (p = 0.69). No significant associations with other inflammation markers were seen. To replicate and assess age differences, data (including TNF-α levels and HF-HRV determined from 11-minutes of ECG recordings) from the Midlife in the United States Study (MIDUS) 2 and Refresher Biomarker Projects (N = 1,664, Mage=55.4) were analyzed. Among adults ≥60 years, the negative HF-HRV–TNF-α association was evident for Black/Black Biracial participants (N = 80, β=–0.280, p = 0.008) but not White participants (N = 474, p = 0.15) in adjusted regression models. For those < 60(years), this association was evident for White participants (N = 703, β=–0.081, p = 0.037) but not for Black/Black Biracial participants (N = 199, p = 0.85). These findings suggest race- and age-specific patterns in the vagal regulation of inflammation. This could have important implications for understanding and addressing cardiovascular health disparities, such as suggesting that interventions targeting HRV may be particularly efficacious among Black individuals in later life.

